# Understanding the Residence Time Distribution in a Transient Inline Spiking System: Modeling, Experiments, and Simulations

**DOI:** 10.3390/membranes13040375

**Published:** 2023-03-25

**Authors:** Minsun Hwang, Junsuk Wang, Seon Yeop Jung

**Affiliations:** Department of Chemical Engineering, Dankook University, Yongin-si 16890, Gyeonggi-do, Republic of Korea; minsun@dankook.ac.kr (M.H.); 32182731@dankook.ac.kr (J.W.)

**Keywords:** continuous bioprocessing, inline spiking, transport phenomena, computational fluid dynamics, viral clearance

## Abstract

A transient inline spiking system is a promising tool for evaluating the performance of a virus filter in continuous operation. For better implementation of the system, we performed a systematic analysis to understand the residence time distribution (RTD) of inert tracers in the system. We aimed to understand the RTD of a salt spike, not retained onto or within the membrane pore, to focus on its mixing and spreading within the processing units. A concentrated NaCl solution was spiked into a feed stream as the spiking duration ($t_{spike}$) was varied from 1 to 40 min. A static mixer was employed to mix the salt spike with the feed stream, which then passed through a single-layered nylon membrane inserted in a filter holder. The RTD curve was obtained by measuring the conductivity of the collected samples. An analytical model, the PFR-2CSTR model, was employed to predict the outlet concentration from the system. The slope and peak of the RTD curves were well-aligned with the experimental findings when $\tau_{PFR}$ = 4.3 min, $\tau_{CSTR1}$ = 4.1 min, and $\tau_{CSTR2}$ = 1.0 min. CFD simulations were performed to describe the flow and transport of the inert tracers through the static mixer and the membrane filter. The RTD curve spanned more than 30 min, much longer than $t_{spike}$, since solutes were dispersed within processing units. The flow characteristics in each processing unit correlated with the RTD curves. Our detailed analysis of the transient inline spiking system would be helpful for implementing this protocol in continuous bioprocessing.

## 1. Introduction

In response to the coronavirus disease 2019 (COVID-19) pandemic, biopharmaceutical industries are being urged to transform from traditional batch processing into continuous processing for rapid and robust production of biopharmaceuticals. In continuous bioprocessing, streams of raw materials are continuously fed and processed without the use of intermediate holding tanks. This decreases processing time, minimizes manufacturing costs, reduces facility footprints, and enhances product quality and affordability [1,2,3,4]. Although tremendous efforts have been made to develop technological solutions for continuous bioprocessing, its implementation has been significantly delayed, especially in downstream bioprocessing, as the validity and feasibility of a given technology have to be demonstrated prior to commercial operation [4]. To realize the continuous manufacturing of biopharmaceuticals, further fundamental development in the downstream processing technology is essential [5].

Virus filtration is an essential step in the downstream bioprocessing, where virus particles are removed by size exclusion either by the membrane surface or by the internal pore [6]. Virus filters are typically located at several processing points to ensure viral safety within the process stream. They are considered robust and reliable units in operation for virus removal as their performance is less influenced by the physicochemical properties of the virus types [7]. A virus spiking procedure is widely used to evaluate the performance of a virus filter, where a given amount of virus solution is spiked, and its concentration is assayed to calculate the total viral clearance [7,8]. For its application in continuous downstream processing, an inline spiking method, where the virus spike is challenged at the location of interest (e.g., upstream of the virus filter, has been utilized in previous studies [6,9]. Bohonak et al. investigated the viral clearance of virus filtration step using both decoupled and inline spiking methods [6]. Malakian and Jung et al. demonstrated a transient inline spiking system and demonstrated its robustness upon multiple virus challenges [9]. These studies identified the usefulness of the inline spiking method; however, the spike characteristics have not yet been systematically studied.

Computational fluid dynamics (CFD) simulations are useful to understand hydrodynamics and mass transfer in the unit operation of chemical and biological processes. In the field of downstream processing, several CFD studies have been performed. David et al. performed a series of CFD simulations of a continuous low pH viral inactivation using a coiled flow inverter to determine the pH level distribution for optimizing the chemical dose [10,11]. Ghosh et al. developed a CFD model to investigate the flow characteristics and protein breakthrough performance in membrane chromatography capsules [12,13]. Jung et al. carried out CFD simulations to represent the breakthrough of ribonuclease A and α-chymotrypsin in a small-scale depth filter module by considering their binding to the filter medium [14]. Similarly to these examples, CFD simulation would be helpful by providing beneficial knowledge on the transient inline spiking system.

In this study, we attempt to characterize the residence time distribution (RTD) curve obtained from a transient inline spiking system by performing modeling, experiments, and simulations. We used a salt spike to focus on the mixing and spreading of the solute within the processing units without any retention issues. A lab-scale transient spiking system was constructed, where a salt spike was injected using a syringe pump. It was mixed with the feed stream through an inline static mixer. It then passed through a single-layered nylon membrane placed in a 25 mm filter holder. The spiking duration ($t_{spike}$) was varied from 1 to 40 min to determine its effect on the shape of the RTD curve. The RTD curve was obtained by measuring the electrical conductivity of the collected samples. A mathematical model, a PFR-2CSTR model, was developed from the mass balance of the spiked solute, postulating the process stream as a combination of a plug flow reactor (PFR) and two continuous stirred-tank reactors (CSTR). To deepen our understanding of the RTD from the transient inline spiking system, CFD simulations were performed in the processing units, namely the static mixer and the filter holder. Flow characteristics and the progress of mixing were analyzed in the static mixer domain to check whether the salt spike is well mixed into the feed stream. The transport of spiked solute under the steady-state flow field within the filter holder was analyzed. The RTD curves obtained from the CFD simulation were compared with those from the experiments and the PFR-2CSTR models.

## 2. Experimental

### 2.1. Transient Inline Spiking System

**Figure 1:**
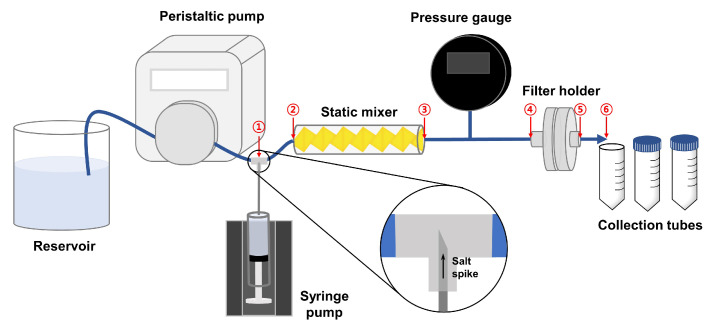
Schematic of the inline spiking system. The syringe needle penetrates the bottom of the tee to directly discharge the salt spike at the center of the feed stream. The circled numbers indicate the processing point to measure the holdup time.

Figure 1 is a schematic representation of the transient inline spiking system. Deionized water in the reservoir was pumped into the system at a constant flow rate of 0.41 mL/min via a peristaltic pump (Masterflex, Gelsenkirchen, Germany). The electrical conductivity of the deionized water was measured at 0.49 ± 0.06 μS/cm using a benchtop conductivity meter (CON700, Eutech Instruments, Singapore). Sodium chloride (NaCl > 99.5 purity, Sigma-Aldrich, St. Louis, MO, USA) was purchased, from which a 1 M NaCl aqueous solution was prepared as the spiking solution. A few milliliters of the spiking solution were loaded into the syringe. A rubber-capped tee was placed behind the peristaltic pump. The spiking solution was discharged at the center region of the flow stream by stabbing the syringe needle into the bottom of the tee. The spiking solution in the syringe was spiked at a 1/10 of feed flow rate, 0.041 mL/min using a syringe pump (Chemyx, Stafford, TX, USA) for a specific spiking duration (1, 5, and 40 min). A static mixer (Part 3/16-12P, Koflo Corp., Cary, IL, USA) was placed after the tee to completely mix the spiking solution with the feed solution. It has a diameter of 4.76 mm, a length of 60.325 mm, and a thickness of 1.0 mm and is composed of 12 mixing elements. The mixed solution was filtered through a nylon membrane (GVS S.p.A., Zona Industriale, Italy) of 5 μm pore size, located in a 25 mm polypropylene filter holder (Advantec, Tokyo, Japan). A nylon membrane that would not retain the NaCl solute was selected since we are focusing on the RTD of the non-interacting solute in the filtration system in this study. A stabilized pressure drop (∼1380 Pa) was observed, indicating no air bubbles were trapped within the process stream. The effluent was collected every 2 min. A sufficient volume of each sample was obtained to ensure a reliable concentration value was measured. A flow pulsation of a few seconds was provided by the peristaltic pump, which is negligible because it is much shorter than the time for sample collection.

Table 1 summarizes the holdup time measurement in each interval. The whole processing stream was first emptied, and the time taken for the flow at a constant flow rate of 0.41 mL/min to pass through each processing point was measured. The longest holdup time was measured in the static mixer (=2.18 ± 0.29 min). The largest standard deviation in the holdup time measurement was found in the filter holder (=1.05 ± 0.57 min). The other intervals, corresponding to the tubing region, showed a relatively small standard deviation in the holdup time. We will discuss these holdup time measurements with the choice of the space time used in our PFR-2CSTR model.

### 2.2. Concentration Easurement

We collected ∼0.8 mL of the sample every two minutes, corresponding to several drops of the effluent. The weight of collected solutions was calculated as the difference between empty tubes and collection tubes after the experiment. Solution conductivity was averaged by triplicating measurements with each solution diluted to 25 mL to be able to locate the probe of the conductivity meter. The concentration of the solution was determined from the concentration–conductivity calibration curve of the NaCl solution. Each concentration of the solution was divided by the concentration when the spiked solute was completely mixed. In this study, since the spiking solution was spiked at 1/10 of the feed flow rate, the concentration at the completely mixed state was $\gamma c_{spike}$, where $c_{spike}$ = concentration of the spiking solution and γ = spiking ratio = 0.1/1.1.

## 3. Modeling and Simulation

### 3.1. Modeling

We assumed that the experimental system could be postulated as a combination of one PFR and two CSTRs. From the initiation of the spike, the inlet concentration of the solute fed into the reactor, $c_{in}$, was described in the rectangular form as below:

(1) $$c_{in}=\left\{\begin{array}{ccc} \gamma c_{spike} & 0\leq t\leq t_{spike}\\ 0 & t>t_{spike} & , \end{array}\right.$$

where *t* is time, and $t_{spike}$ is the spiking duration in the experiment. A process stream with a time lag in the tubing and some portion of processing units could be described by a PFR:

(2) $$c_{0}=c_{in}H\left(t-\tau_{PFR}\right),$$

where $c_{0}$ is the salt concentration flowing out of PFR, H(x) is the Heaviside step function (H(x)=1 when x>0 and H(x)=0 when x≤0), $\tau_{PFR}$ is the space time of PFR ($\tau_{PFR}=V_{PFR}/v$), and *v* is flow velocity. On the other hand, the spreading and mixing of solute occurring in the static mixer, filter holder, and tubing were lumped together as two CSTRs in our model:

(3) $$c_{0}-c_{1}=\tau_{1}\frac{dc_{1}}{dt},$$

(4) $$c_{1}-c_{2}=\tau_{2}\frac{dc_{2}}{dt},$$

where $\tau_{1}=V_{1}/\nu$ and $\tau_{2}=V_{2}/\nu$ are the space time of each CSTR, $c_{1}$ and $c_{2}$ are the salt concentration in each reactor, and $V_{1}$ and $V_{2}$ are the volume of each reactor. The model equation for the outlet concentration ($c_{out}$) from the PFR-2CSTR system was evaluated by combining Equations (1)–(4):

(5) $$\begin{array}{c} \begin{array}{ccc} c_{out} & = & c_{in}[\left(1+\frac{\tau_{1}}{\tau_{2}-\tau_{1}}e^{-\frac{t}{\tau_{1}}}-\left(1+\frac{\tau_{1}}{\tau_{2}-\tau_{1}}\right)e^{-\frac{t}{\tau_{2}}}\right)H\left(t-\tau_{PFR}\right)\\ \; & \; & -\left(1+\frac{\tau_{1}}{\tau_{2}-\tau_{1}}e^{-\frac{t-t_{spike}}{\tau_{1}}}-\left(1+\frac{\tau_{1}}{\tau_{2}-\tau_{1}}\right)e^{-\frac{t-t_{spike}}{\tau_{2}}}\right)H\left(t-\tau_{PFR}-t_{spike}\right)]. \end{array} \end{array}$$

### 3.2. CFD Simulation

Although the outlet concentration ($c_{out}$) could be modeled by Equation (5), the spatiotemporal distribution of inert tracers within the processing units (i.e., static mixer and filter holder) cannot be obtained from the model. To deepen our understanding of the RTD of inert tracers, a series of CFD simulations were performed. While it would be ideal to perform simulations describing the whole processing stream, our computational resources were limited. Therefore, we decided to perform CFD simulations in the static mixer and filter holder separately, since the remaining tubing regions would act as PFRs.

**Figure 2:**
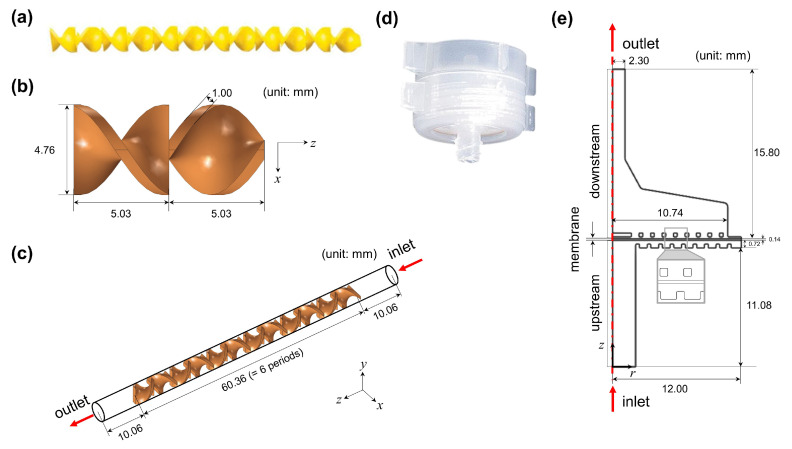
Processing units used in the transient inline spiking system and simulation domains for CFD simulation: (**a**) the geometry of the static mixer, (**b**) the periodic unit of the static mixer, and (**c**) the static mixer (SM) simulation domain in three dimensions composed of six periodic units of the static mixer and two buffer regions at the inlet and outlet. (**d**) The geometry of the filter holder, and (**e**) the filter holder (FH) in two dimensions with axisymmetry. A red single dash-dotted line in the simulation domain of the filter holder represents the axis of rotation.

We constructed two separate simulations in the static mixer domain and the filter holder domain. Figure 2a depicts the static mixer used in the experimental system. The periodic unit of the static mixer is shown in Figure 2b, where a mixing element is orthogonally connected to the other mixing element. The dimensions are obtained by measuring the static mixer used in the experiment. CFD simulation in the static mixer channel was performed to check whether the spiked species would be mixed into the process stream. Figure 2c is the 3D simulation domain with six periodic units of the static mixer and two buffer regions. The number of periodic units is the same as that used in the experiment. Two buffer regions are inserted at the inlet and the outlet of the mixer channel to avoid any unwanted numerical artifacts if the flow is directly influenced by the mixing element near the inlet and the outlet. Figure 2d shows the filter holder used in the experiment. Figure 2e describes the 2D axisymmetric simulation domain of the filter holder, composed of the upstream, downstream, and membrane regions. In the upstream region, fluid is fed inside the filter holder, while in the downstream region, fluid passes through the outlet via the membrane. Table 2 summarizes the parameters used in the simulations.

The velocity field within the static mixer (or filter holder) was evaluated by solving the steady-state Navier–Stokes equation and the continuity equation for an incompressible fluid in laminar flow regime as below:

(6) $$\rho \left(u\cdot ▿u\right)=-▿p+\mu {▿}^{2}u,$$

(7) ▿·u=0,

where ρ is the fluid density, u is the fluid velocity vector, μ is the fluid viscosity, and p is the pressure. Here, ρ and μ are known to be a function of the salt concentration [15,16]. As the salt concentration is varied from 0 to 1 M, the Reynolds number (Re) in the static mixer domain, defined as Re=ρuD/μ where *D* is the diameter of the circular inlet, changes from 1.84 to 1.90. Similarly, in the filter holder domain, Re changes from 1.89 to 1.94. The flow field in each processing unit would not be significantly changed by this difference in Re. Hence, the density and viscosity of the working fluid are assumed to be constant as ρ=997.5 kg/m^3^ and μ=0.898 mPa·s at the values of 0.1 M salt concentration at 25 °C (see Figure S1 in Supporting Information). Readers may refer to previous numerical studies on laminar mixing in static mixers [17,18,19,20].

In the membrane region of the filter holder domain, the governing equation of Darcy’s law was solved as described below:

(8) $$▿\cdot \left(\rho u\right)=Q_{m},$$

(9) $$u=-\frac{\kappa }{\mu }▿p,$$

where $Q_{m}$ is the mass flux of fluid through the membrane, and κ is permeability. In the experiment, some regions of the membrane were compressed when the filter holder was screwed together. However, we neglected the compression of the membrane because the RTD of tracers would be much more dependent on their spreading and mixing inside the processing units compared to their transport within the membrane.

Boundary conditions for the flow were as follows. For each processing unit (i.e., the static mixer or the filter holder), a fully developed flow field in the circular channel ($u\left(z\right)=2{\bar{u}}_{in}\left(1-\left(4\left(x^{2}+y^{2}\right)/D^{2}\right)\right),u\left(x\right)=u\left(y\right)=0$, where ${\bar{u}}_{in}$ is the average inlet velocity and *D* is the diameter of the processing unit) was assumed at the inlet. At the outlet, a constant pressure boundary condition (*p* = 0) was assigned. No-slip boundary condition (***u*** = 0) was applied to the remaining solid boundaries.

After the flow field was obtained in each processing unit, the mass transfer of the solute inside the static mixer (or filter holder) over time was evaluated by solving the transient mass transfer equation as below:

(10) $$\frac{\partial c}{\partial t}+▿\cdot J+u\cdot ▿c=0,$$

(11) J=−D▿c,

where *c* is salt concentration, *J* is the mole flux of salt, *D* is diffusion coefficient (=1.68 × 1$0^{-9}$ m/s^2^, an average of the diffusion coefficients of Na+ and Cl− [21]). As the solute was treated as an inert tracer, a reaction term was not considered in this equation.

Boundary conditions for the mass transfer of the solute were assigned as follows: At the inlet of the static mixer channel, a circular region corresponding to the needle tip of the syringe with a diameter of $D_{s,SM}$ = 0.337 mm was located. The inlet concentration at the needle tip was set to $c_{in,SM}=c_{spike}=1$ M, where a concentrated solute was introduced into the static mixer channel. At the remaining boundaries of the static mixer, including the outlet, the diffusive mass flux was assigned as 0 (n·D▿c=0), a typical boundary condition for them. In the filter holder domain, we assumed that the solute at the completely mixed state was introduced at the inlet. This would be checked by performing the CFD simulation in the static mixer domain. With this assumption, the concentration at the inlet of the filter holder was assigned as $c_{in,FH}=\gamma c_{spike}$ during a spike ($t_{0}\leq t\leq t_{0}+t_{spike}$, where $t_{0}$ is the time when the completely mixed stream of the feed solution reached the inlet port of the filter holder) and then changed to c=0 after a spike ($t>t_{0}+t_{spike}$). The boundary condition, n·D▿c=0 was imposed at the outlet and the solid boundaries of the filter holder.

**Figure 3:**
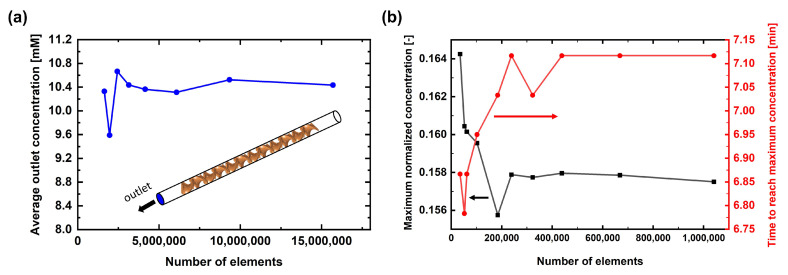
Mesh convergence test results for the CFD simulations of (**a**) the static mixer and (**b**) the filter holder.

The commercial CFD software program COMSOL Multiphysics 5.4 (COMSOL, Inc., Burlington, MA, USA), based on the finite element method (FEM), was used in this study. Equations (6) and (7) were solved to determine the steady-state flow field in the static mixer domain, while Equations (6)–(9) were solved in a coupled manner to determine the steady-state flow field in the filter holder domain. When the steady-state velocity profile was obtained, transient simulation was conducted by solving Equations (10) and (11) to determine the spatiotemporal distribution of the solute in the simulation domain. Figure 3 shows the mesh convergence test conducted both in the static mixer and filter holder domains. In the 3D static mixer domain, 5,405,839 computational elements were used with an average element quality of 0.67. Most of the elements were tetrahedra (=4,920,623), while some were prisms (=475,020) and pyramids (=10,196), as the boundary layer elements were placed near the channel wall. In the 2D axisymmetric filter holder domain, 675,470 computational elements were used to perform the simulation with an average element quality of 0.90. Most of the elements were triangles (=663,114), while some quadratic elements (=12,356) were used to place boundary layers near the solid boundaries. A linear scheme (P1/P1) was used both for the discretization of fluid velocity and pressure, which is the default discretization scheme of the software for the flow simulation under a laminar regime. An iterative solver used for a large sparse linear system, GMRES (Generalized Minimal RESidual method), was used for the simulation performed in the static mixer domain, while a parallel sparse direct solver, MUMPS (MUltifrontal Massively Parallel Sparse direct Solver), was used for the simulation in the filter holder domain. The solution was considered to be converged when the relative tolerance became less than $1\times 10^{-5}$. A workstation was used for the simulation in the 3D static mixer domain (Intel(R) Xeon(R) CPU E5–2687 (3.1 GHz), 512 GB memory), and a high-performance computer (11th Gen Intel(R) Core(TM), CPU i5-11600K (3.91 GHz), 64 GB memory) was used for the simulation in the 2D axisymmetric filter holder domain.

## 4. Results and Discussion

### 4.1. RTD Curve

**Figure 4:**
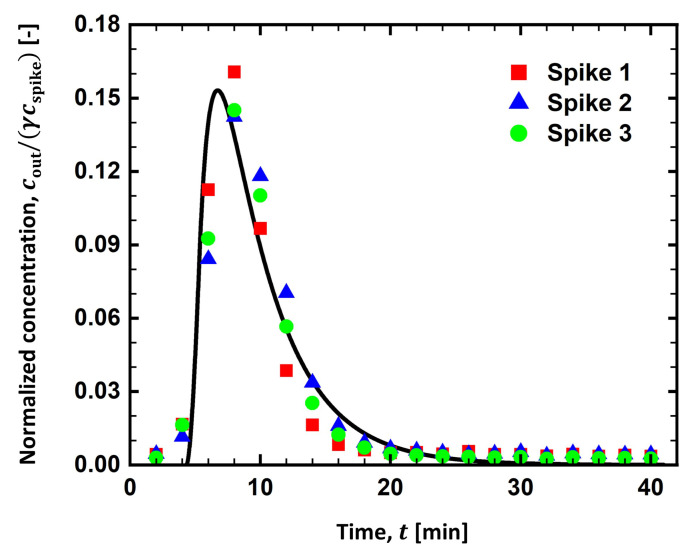
RTD curve from three repeated runs of the salt spiking experiments. The feed flow rate was 0.41 mL/min, and the spiking duration was 1 min at a flow rate of 0.041 mL/min.

Figure 4 shows the dimensionless salt concentration ($c_{out}/\left(\gamma c_{spike}\right)$) at the outlet from three repeated runs of inline spiking experiments when $t_{spike}$ = 1 min. Each experiment showed well-reproduced RTD curves overlapping each other. While salt is spiked for 1 min, its elution started to be detected at around 3 min from the beginning of the experiment, indicating the time needed for the salt to pass through the process stream. Compared to $t_{spike}$, the RTD curve spanned a long time (∼30 min), as the solute was dispersed within operating units (i.e., static mixer, filter holder, and tubing). This significant band broadening is generally not desirable since it takes a longer time to obtain an RTD curve and to evaluate the performance of the filter. The RTD curve sharply increased from 4 to 8 min, then gradually decreased, and finally diminished at around 24 min. The solid curve is the prediction of the PFR-2CSTR model. We manually changed the values of the space time of each reactor ($\tau_{PFR}$, $\tau_{CSTR1}$, and $\tau_{CSTR2}$) and found a good agreement when $\tau_{PFR}$ = 4.3 min, $\tau_{CSTR1}$ = 4.1 min, and $\tau_{CSTR2}$ = 1.0 min. It would be helpful to discuss the space time with the measured holdup time in Table 1. The total holdup time through the tubing, considered to behave as a PFR, was 2.30±0.10 min, smaller than $\tau_{PFR}$. It is inferred that a fractional volume of the static mixer and the filter holder worked as additional PFR for the fluid. On the other hand, the total holdup time through the static mixer and the filter holder was measured as 3.23±0.86 min, smaller than $\tau_{CSTR1}$ and larger than $\tau_{CSTR2}$. From this analysis, a tubing is found not to behave as an ideal PFR. Some fraction of tubing would be added as an additional volume of the CSTRs in the PFR-2CSTR model.

**Figure 5:**
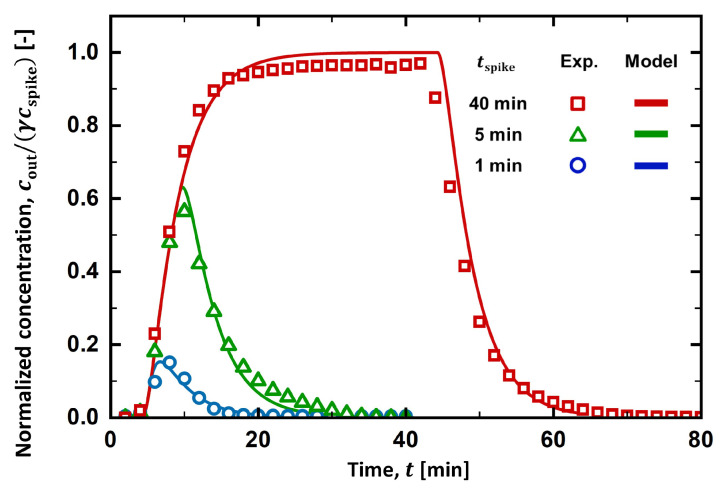
Effect of spiking duration on the RTD curves when the feed flow rate is 0.41 mL/min.

As depicted in Figure 5, $t_{spike}$ was varied from 1 to 40 min to determine its effect on the RTD curves. As $t_{spike}$ increased to 5 min, the peak height increased to $c_{out}/\left(\gamma c_{spike}\right)∼0.6$, and its location shifted to ∼10 min. The area under the RTD curve also increased, meaning more salt was spiked into the stream. When $t_{spike}$ = 40 min, the RTD curve sharply increased for ∼20 min then reached a plateau region, where its maximum value ($c_{out}/\left(\gamma c_{spike}\right)∼0.97$) was found. While the salt spiking was maintained, the stream was completely mixed with the spiking solution, yielding a plateau of the RTD curve at the maximum value. When the salt spiking ceased, however, the salt concentration at the outlet started to decrease as the solute present in the process stream was emptied by the feed stream. It took more than 20 min for the solute to be removed from the processing units.

The prediction of the PFR-2CSTR model was in excellent accordance with the RTD curves obtained at all $t_{spike}$ investigated in the experiment, while the space time ($\tau_{PFR}$, $\tau_{CSTR1}$, and $\tau_{CSTR2}$) remained unchanged. Before their peaks, RTD curves evaluated at different $t_{spike}$ overlapped each other at the initial rise in the salt concentration. When $t_{spike}$ = 1 and 5 min, the RTD curves dramatically decreased as the net outflow of the solute from the system was found. When $t_{spike}$ = 40 min, however, a plateau region developed, and $c_{out}/\left(\gamma c_{spike}\right)$ approached 1. This is because the spiking was maintained as $t_{spike}$ reached its maximum value. When $t_{spike}$ = 40 min, $c_{out}/\left(\gamma c_{spike}\right)$ reached 1 in the model; however, it just reached ∼0.97 in the experiment. In the experiment, spiked salts would not be fully recovered at the outlet because a small fraction of solute would remain in dead space within the process stream or be absorbed in an unknown spot.

### 4.2. Static Mixer

**Figure 6:**
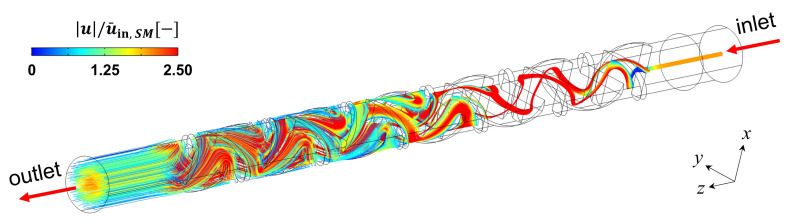
Streamlines evaluated from the center of the inlet where the salt spike was discharged through the syringe needle. The color of the streamline represents the velocity magnitude divided by ${\bar{u}}_{in,SM}$.

We performed a CFD simulation in the static mixer domain to check whether the spiked solute from the tip of the syringe needle would be sufficiently mixed into the feed stream by the static mixer. Figure 6 represents the streamlines whose starting points are located at the tip of the syringe needle. The number of starting points was 1000. Streamlines started from the center of the channel, and they followed similar paths in the first and second mixing periods. However, their endpoints became dispersed at the outlet by the presence of the static mixer. Our static mixer is a Kenics mixer, where chaotic advection would be generated for a laminar flow regime [19,20,22,23]. The number of mixing periods was found to be sufficient to distribute the streamlines initially starting from the narrow region. This means that the spiked solute would be satisfactorily mixed with the processing stream.

**Figure 7:**
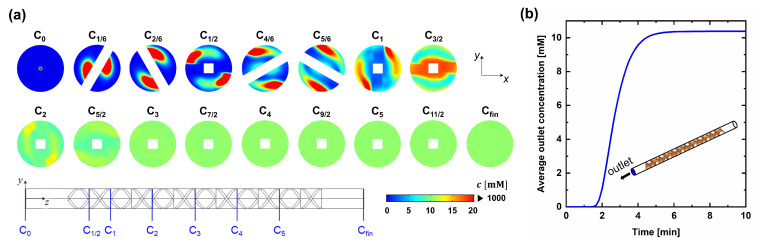
Concentration distribution in the static mixer. (**a**) Concentration distribution ($C_{n}$) at the cross-section of the n-th mixing period. When c ≥ 20 mM, the color of the contour is represented in red because the maximum concentration is much larger than the concentration at the mixed state. (**b**) The RTD curve in the static mixer when the concentration at the tip of the syringe needle is kept constant at 1 M.

Figure 7a is the concentration distribution ($C_{n}$) at the cross-section of the n-th mixing period. It was obtained by the solution of Equation (10) at the steady state (∂/∂t=0), corresponding to the case when the concentration at the tip of the syringe needle was kept constant at 1 M. At $C_{0}$, the concentration at the central region was 1 M, where the tip of the syringe needle was assumed to be placed. From  $C_{1/6}$ to $C_{1/2}$, the stream was divided by the twisted plate and the flow in each stream rotated clockwise. From $C_{4/6}$ to $C_{1}$, on the other hand, the stream was divided by another twisted plate and the flow in each stream rotated counter-clockwise. Stretching and folding of the fluid were induced through this process, and chaotic advection was triggered. As the number of mixing periods increased to 6, the resulting concentration distribution at the cross-section became more homogeneous (see $C_{fin}$). In the experiment, six mixing periods were used, which is sufficient for mixing the spiked solute into the feed stream, as found in the simulation results. Figure 7b is the RTD curve of the solute obtained from the transient solution of Equation (10) (Here, ∂/∂t≠0). Solute started to come at ∼1.5 min, and the average outlet concentration gradually increased to reach its maximum value at ∼5 min. It is inferred that the static mixer fractionally works as a PFR, providing a time lag for the solute to be detected at the outlet. Spreading of the solute was also found, as witnessed by the time delay of ∼3.5 min for the average outlet concentration to reach its maximum value.

### 4.3. Filter Holder

**Figure 8:**
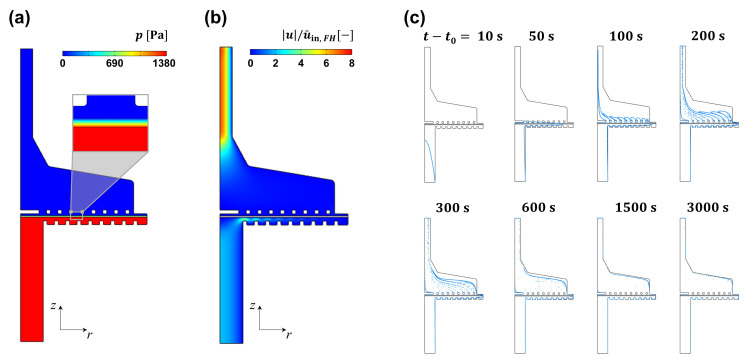
(**a**) Pressure field within the filter holder, (**b**) velocity magnitude in the filter holder, and (**c**) trajectory of passive tracers under the flow that develops within the filter holder, where $t_{0}$ is the time when the completely mixed stream of the feed solution reaches the inlet port of the filter holder.

From the simulation conducted in the static mixer domain, we could find that the spiked solute would be well mixed into the processing stream and then provided to the filter holder. From this finding, we could confirm that the assumption for the boundary condition at the inlet of the filter holder, $c_{in}=\gamma c_{spike}$, is reasonable. Hence, we could move on to the CFD simulation in the filter holder domain. Figure 8a is the pressure field developed within the filter holder calculated from the CFD simulation performed in the filter holder domain. Upstream of the filter holder, the pressure value was 1380 Pa, identical to the pressure value in the experiment. The pressure level gradually decreased along the depth of the membrane, as the instantaneous discharge was related to the pressure drop in that distance in Darcy’s law. Pressure in the downstream region was almost zero because the pressure at the outlet was set to zero and the pressure drop in the open channel (i.e., downstream region) was negligible. Figure 8b represents the magnitude of the fluid velocity divided by the inlet velocity into the filter holder. At the inlet port, the fluid velocity was higher near the axis of rotation, because a parabolic flow field, the fully developed laminar flow in a circular channel, was provided. As the flow reached the membrane region, it was spread in the radial direction. It took time for the flow to pass through the membrane with a significant pressure drop. At the outlet port, the the fluid velocity became higher again near the axis of rotation, as a parabolic flow field developed in this region. The velocity magnitude was higher at the outlet port than at the inlet port because the outlet port has a smaller diameter than the inlet port. Figure 8c represents the results from particle tracing analysis performed to visualize the transport of passive tracers by the flow field in the filter holder. The trajectories of the passive tracer were described by the momentum balance equation of massless particles.

(12) $$\frac{dq}{dt}=u,$$

where q is the displacement vector. A tracing particle was presumed to be chargeless and passively transported by the flow. The trajectories were determined with a total of 20,000 particles located at the inlet of the filter holder. Each snapshot was taken at the time represented after the completely mixed stream of the feed solution reached the inlet port of the filter holder (=$t-t_{0}$), where $t_{0}$ is the time when the completely mixed stream of the feed solution reaches the inlet port of the filter holder. At $t-t_{0}$ = 10 s, particles were aligned on a parabolic curve, where particles located near the axis of rotation move faster than those located near the solid wall of the inlet port. At $t-t_{0}$ = 50 s, particles were radially transported near the membrane in the upstream region while it took a considerable time to pass through the membrane because of the flow resistance by the membrane. When $t-t_{0}$ = 100 s, particles having passed through the central region of the membrane approached the outlet much quicker than those having passed through the outer region of the membrane. It took a much longer time for particles moving near the solid boundary of the filter holder to reach the outlet, as shown in the snapshots at 600 and 1500 s. From the particle tracing, significant retardation of the solute transport was expected for a solute moving near the solid boundary of the filter holder. It would take a much longer time if the tracers could bind within the process units such as the tubing, surface of the static mixer, and membrane pores.

**Figure 9:**
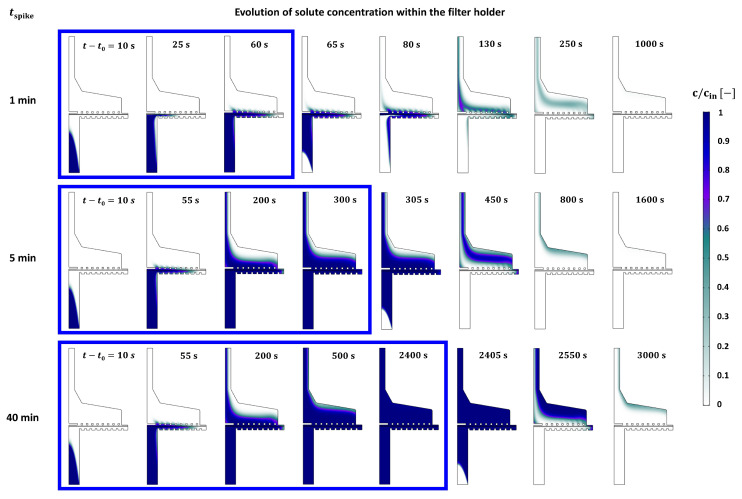
Evolution of solute concentration within the filter holder at varying spiking duration. Blue boxes represent the time duration before the end of each spike.

Figure 9 represents the evolution of solute transport in the filter holder. As discussed in the particle tracing analysis, solute near the axis of rotation approached the membrane much faster than that near the solid boundary of the inlet port. When $t_{spike}$ = 1 min, the spiking ceased at $t-t_{0}$ = 60 s while the solute at the central region just passed through the membrane. After the spiking ceased, the concentration of the feed solution became 0, while the spiked solute was being pushed to the outlet. Having passed the outer region of the membrane, it took much longer time for the solute to reach the outlet compared with that passing through the central region of the membrane. As time progressed, the concentration at the central region became lower, and the spiked solution at the outer region was eluted. Similar elution behavior was found as $t_{spike}$ increased to 5 and 40 min. When $t_{spike}$ = 5 min, the solute was eluted even before the end of spiking (see the snapshot when $t-t_{0}$ = 200 s), while when the spike ceased at $t-t_{0}$ = 300 s, the concentration at the outlet became almost saturated. When $t_{spike}$ = 40 min, the whole volume of the filter holder became fully saturated before the end of spiking. It means that $c_{out}/\left(\gamma c_{spike}\right)$ would remain saturated when the filter holder was saturated by the solute. After the spike ceased, the remaining salt started to be eluted as the pure feed solution was injected into the system. From this analysis, it can be drawn that a long $t_{spike}$ is required for the solute to be fully saturated within a processing unit. This means that the holdup volume of the processing unit should be minimized to reduce the band broadening when applying the inline spiking system to actual continuous processing.

**Figure 10:**
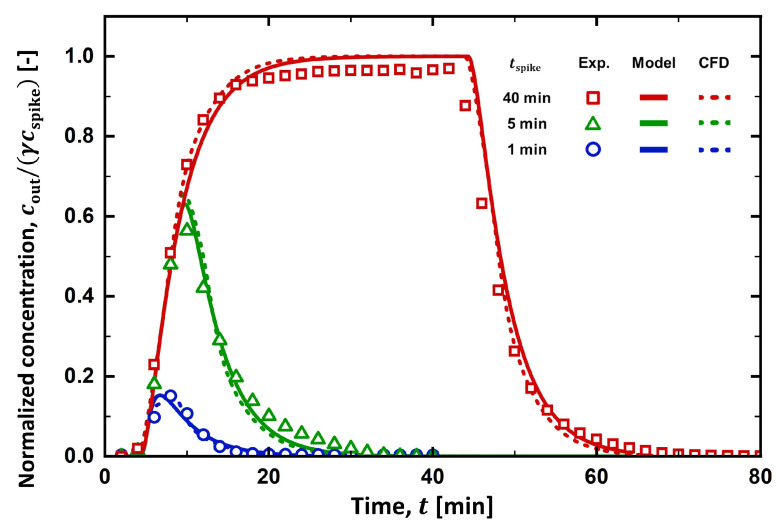
The RTD curves from the CFD simulation compared to those from the experiments and the PFR-2CSTR model.

Figure 10 summarizes the RTD curves obtained from the experiments, the PFR-2CSTR model, and the CFD simulations. The RTD curve from the CFD simulation was prepared by evaluating the average salt concentration at the outlet with time. When $t_{0}$ was set to 2.7 min, the RTD curve from the CFD simulation aligned with the RTD curves obtained from both the experiment and the PFR-2CSTR model. The reason why $t_{0}$ (=2.7 min) had to be shorter than $\tau_{PFR}$ (=4.3 min) to obtain the well-matched RTD curves is unclear. When $t_{spike}$ = 1 min, however, the CFD prediction at the peak concentration slightly deviated from the prediction of the PFR-2CSTR model. As shown in Figure 9, solute was not completely mixed within the filter holder. This incomplete mixing became distinct at a short spiking duration. As $t_{spike}$ increased, the peak concentration was less sensitive to the mixing in the holder as the outlet concentration was saturated to its maximum value, $c_{out}/\left(\gamma c_{spike}\right)$. It was inferred that the spiking duration should be long enough to understand the flow system and to be less sensitive to the solute mixing within the filter holder.

We conducted an additional run of the CFD simulation for a virus filtration membrane. From our previous study [9], the transmembrane pressure through the Viresolve(R) Pro virus filtration membrane (Merck, Rahway, NJ, USA) was ∼1380 Pa. The flow characteristics and RTD curves from the filter holder equipped with either the nylon membrane or the virus filtration membrane were depicted in Figure S2 in Supporting Information. Although the pressure drops through each membrane were different, the flow field in each filter holder was almost identical. This means that our current study of the inline spiking system with the nylon membrane is representative of the system with the virus filtration membrane, even though they have different pore size distributions.

## 5. Conclusions

In this study, the RTD of non-interacting tracers in a transient inline spiking system was studied by modeling, experiments, and simulations. A lab-scale inline spiking experiment was performed with the use of a static mixer and a filter holder, where a single-layered nylon membrane was inserted. NaCl solution at 1 M was spiked into the processing stream through a syringe needle, and the RTD curve of the spiked solute was obtained by measuring the electrical conductivity of the collected samples. As $t_{spike}$ was varied from 1 to 40 min, the PFR-2CSTR model provided a fair prediction of the RTD curves. The slope and peak of the RTD curves were well represented for a fixed set of space time values ($\tau_{PFR}$ = 4.3 min, $\tau_{CSTR1}$ = 4.1 min, and $\tau_{CSTR2}$ = 1.0 min). They were correlated and discussed with the measured holdup time in each processing unit. A series of CFD simulations were performed to visualize the solute transport in the static mixer domain and in the filter holder domain to understand the RTD curves at varying $t_{spike}$. From the CFD simulation results performed in the static mixer domain, the number of mixing periods used in the experiment was found to be sufficient for mixing the spiked solute into the processing stream. The RTD curves calculated from the CFD simulations performed in the filter holder domain were in good agreement with the experimental findings and the model prediction. Solute having passed the central region of the membrane was more quickly eluted than that which passed the outer region of the membrane. Although there were slight deviations in the CFD simulation and the PFR-2CSTR model, our study helps to understand the breakthrough behavior in the transient inline spiking system. Significant band broadening (∼30 min) was observed from the holdup volume of processing units, requiring a much longer time than the spiking duration to obtain the RTD curve. From this end, one may consider avoiding band broadening when introducing a transient inline spiking method in actual continuous processing.

## Figures and Tables

**Table 1:** Holdup time measurement.

No.	Interval	Holdup Time [min]	Description
1	①–②	0.98 ± 0.02	Tubing (spiking station → static mixer)
2	②–③	2.18 ± 0.29	Static mixer
3	③–④	0.98 ± 0.07	Tubing (static mixer → filter holder)
4	④–⑤	1.05 ± 0.57	Filter holder
5	⑤–⑥	0.34 ± 0.01	Tubing (filter holder → collection tube)

**Table 2:** Simulation parameters.

Static Mixer (SM) Domain			
**Symbol**	**Value**	**Unit**	**Description**
${\bar{u}}_{in,SM}$	$3.8\times 10^{-4}$	m/s	Average inlet velocity into the static mixer
$D_{SM}$	4.76	mm	Diameter of the static mixer
$D_{s,SM}$	0.337	mm	Diameter of the syringe needle
$L_{p,SM}$	10.06	mm	Length of the periodic unit of the static mixer
$h_{SM}$	1.00	mm	Thickness of the static mixer
**Filter Holder (FH) domain**			
**Symbol**	**Value**	**Unit**	**Description**
${\bar{u}}_{in,FH}$	$4.3\times 10^{-4}$	m/s	Average inlet velocity into the filter holder
$D_{i,FH}$	4.3	mm	Diameter of the inlet of the filter holder
$D_{o,FH}$	2.3	mm	Diameter of the outlet of the filter holder
$D_{m}$	24	mm	Diameter of the membrane
$\Delta P_{m}$	1380	Pa	Transmembrane pressure (TMP)
$h_{m}$	140	μm	Membrane thickness
κ	$1.27\times 10^{-15}$	m^2^	Membrane permeability

## Data Availability

Not applicable.

## Supplementary Materials

The following supporting information can be downloaded at: https://www.mdpi.com/article/10.3390/membranes13040375/s1.

## References

[1] Gerstweiler L., Bi J., Middelberg A.P.J. (2021). Continuous downstream bioprocessing for intensified manufacture of biopharmaceuticals and antibodies. Chem. Eng. Sci..

[2] Coffman J., Brower M., Connell-Crowley L., Deldari S., Farid S.S., Horowski B., Patil U., Pollard D., Qadan M., Rose S. (2021). A common framework for integrated and continuous biomanufacturing. Biotechnol. Bioeng..

[3] Khanal O., Lenhoff A.M. (2021). Developments and opportunities in continuous biopharmaceutical manufacturing. mAbs.

[4] Zydney A.L. (2016). Continuous downstream processing for high value biological products: A Review. Biotechnol. Bioeng..

[5] Renate K., Reinhart D. (2016). Advances in recombinant antibody manufacturing. Appl. Microbiol. Biotechnol..

[6] Bohonak D.M., Mehta U., Weiss E.R., Voyta G. (2021). Adapting virus filtration to enable intensified and continuous monoclonal antibody processing. Biotechnol. Prog..

[7] Johnson S.A., Chen S., Bolton G., Chen Q., Lute S., Fisher J., Brorson K. (2022). Virus filtration: A review of current and future practices in bioprocessing. Biotechnol. Bioeng..

[8] Lutz H., Chang W., Blandl T., Ramsey G., Parella J., Fisher J., Gefroh E. (2011). Qualification of a novel inline spiking method for virus filter validation. Biotechnol. Prog..

[9] Malakian A., Jung S.Y., Afzal M.A., Carbrello C., Giglia S., Johnson M., Miller C., Rayfield W., Boenitz D., Cetlin D. (2022). Development of a transient inline spiking system for evaluating virus clearance in continuous bioprocessing—Proof of concept for virus filtration. Biotechnol. Bioeng..

[10] David L., Waldschmidt L.M., Lobedann M., Schembecker G. (2020). Simulation of pH level distribution inside a coiled flow inverter for continuous low pH viral inactivation. Biotechnol. Bioeng..

[11] David L., Bayer M.P., Lobedann M., Schembecker G. (2020). Simulation of continuous low pH viral inactivation inside a coiled flow inverter. Biotechnol. Bioeng..

[12] Ghosh P., Vahedipour K., Lin M., Vogel J.H., Haynes C., von Lieres E. (2013). Computational fluid dynamic simulation of axial and radial flow membrane chromatography: Mechanisms of non-ideality and validation of the zonal rate model. J. Chromatogr. A.

[13] Ghosh P., Vahedipour K., Leuthold M., von Lieres E. (2014). Model-based analysis and quantitative prediction of membrane chromatography: Extreme scale-up from 0.08 mL to 1200 mL. J. Chromatogr. A.

[14] Jung S.Y., Nejatishahidein N., Kim M., Espah Borujeni E., Fernandez-Cerezo L., Roush D.J., Borhan A., Zydney A.L. (2021). Quantitative interpretation of protein breakthrough curves in small-scale depth filter modules for bioprocessing. J. Membr. Sci..

[15] Out D.J.P., Los J.M. (1980). Viscosity of aqueous solutions of univalent electrolytes from 5 to 95 °C. J. Solut. Chem..

[16] Simion A.I., Grigoras C., Rosu A.M., Gavrilă L. (2015). Mathematical modelling of density and viscosity of NaCl aqueous solutions. J. Agroaliment. Process. Technol..

[17] Kang T.G., Singh M.K., Anderson P.D., Meijer H.E.H. (2009). A chaotic serpentine mixer efficient in the creeping flow regime: From design concept to optimization. Microfluid. Nanofluidics.

[18] Meijer H., Singh M., Anderson P. (2012). On the performance of static mixers: A quantitative comparison. Prog. Polym. Sci..

[19] Jung S.Y., Ahn K.H., Kang T.G., Park G.T., Kim S.U. (2018). Chaotic mixing in a barrier-embedded partitioned pipe mixer. AIChE J..

[20] Jung S.Y., Park J.E., Kang T.G., Park J.D. (2022). Flow and mixing analysis of a thixotropic fluid in a barrier-embedded partitioned pipe mixer (BPPM): A numerical study. Int. J. Heat Mass Transf..

[21] Haynes W. (2016). CRC Handbook of Chemistry and Physics.

[22] Ottino J., Crighton D. (1989). The Kinematics of Mixing: Stretching, Chaos, and Transport.

[23] Ghahfarokhi N.J., Bayareh M. (2021). Numerical study of a novel spiral-type micromixer for low Reynolds number regime. Korea Aust. Rheol..

