# Supplementary material for: Understanding the Residence Time Distribution in a Transient Inline Spiking System: Modeling, Experiments, and Simulations
Source: Membranes (Basel). 2023 Mar 25;13(4):375. doi: 10.3390/membranes13040375 (PMC10143522; doi:10.3390/membranes13040375)
Supplement: Supplementary file 1 [file membranes-13-00375-s001.zip › membranes-2290612-supplementary.pdf]

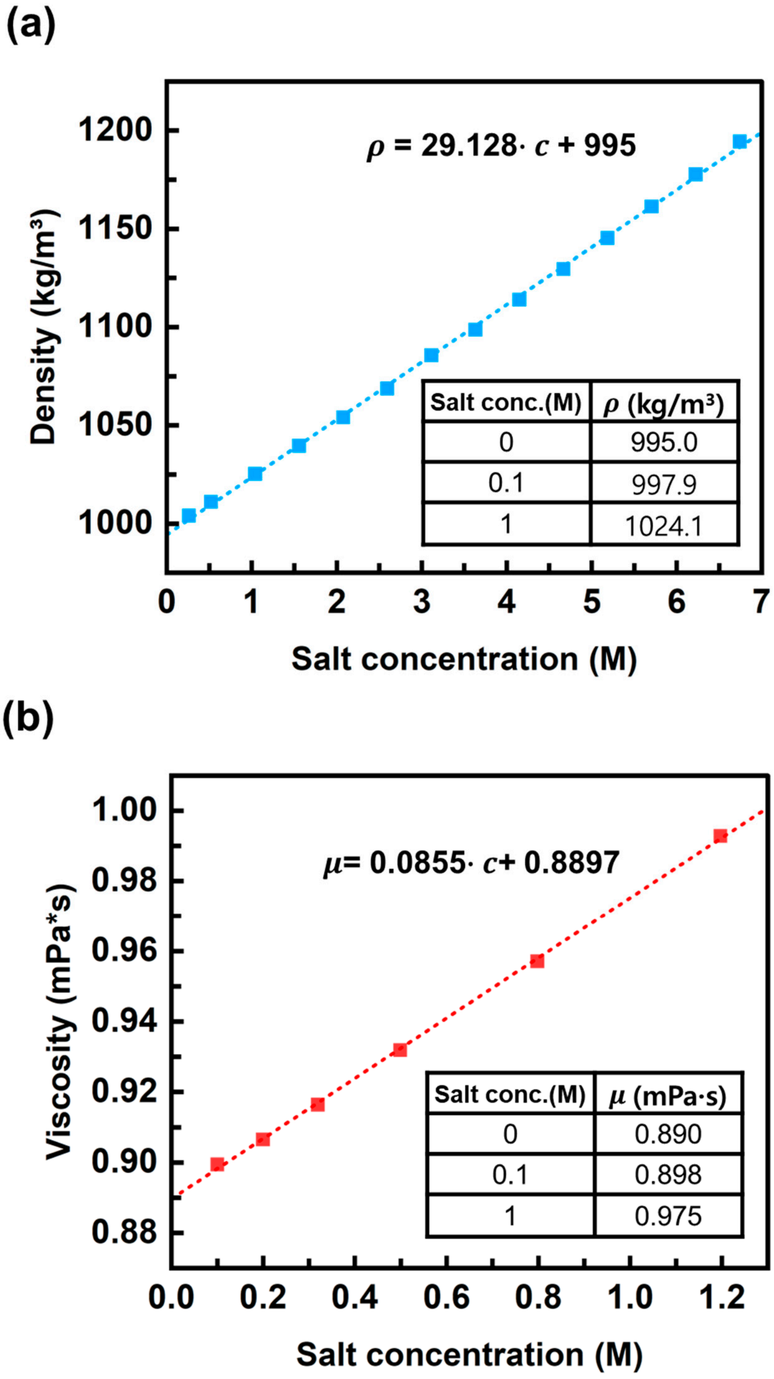

Figure S1. (a) Density and (b) viscosity of the sodium chloride (NaCl) solution at varying salt concentration.

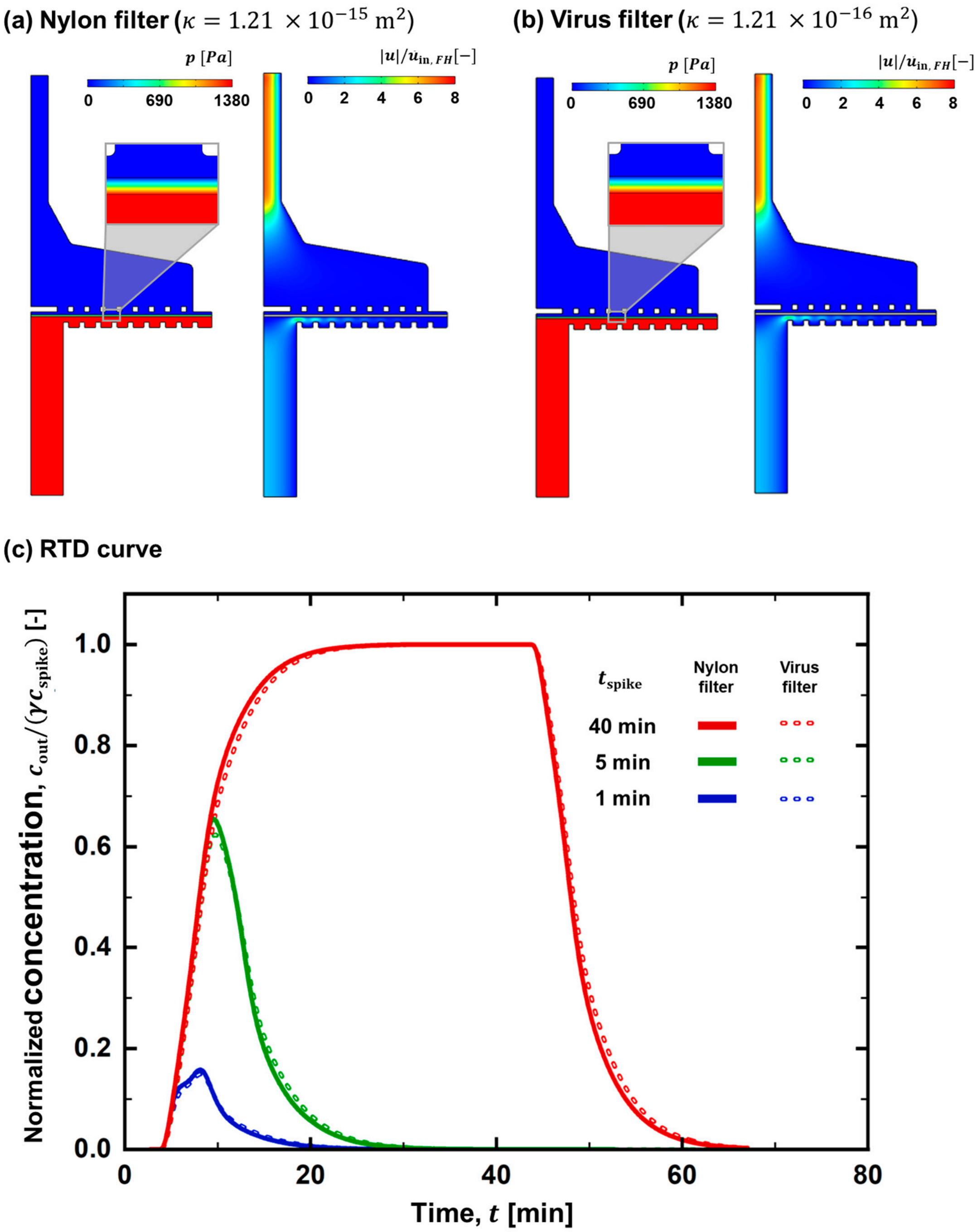

Figure S2. Comparison of the nylon filter and the virus filter: Flow characteristics when either (a) the nylon filter or (b) the virus filter was inserted in the filter holder. (c) RTD curves when either the nylon filter or the virus filter was used at different spiking duration.
